# Multi-Constellation Software-Defined Receiver for Doppler Positioning with LEO Satellites

**DOI:** 10.3390/s20205866

**Published:** 2020-10-16

**Authors:** Farzan Farhangian, René Landry

**Affiliations:** LASSENA Laboratory, Department of Electrical Engineering, Ecole de Technologie Superieure, Montreal, QC H3C-1K3, Canada; renejr.landry@etsmtl.ca

**Keywords:** software-defined radio, LEO satellite, Doppler positioning, navigation, Orbcomm, Iridium, Globalstar, signals of opportunity

## Abstract

A Multi-Constellation Software-Defined Receiver (MC-SDR) is designed and implemented to extract the Doppler measurements of Low Earth Orbit (LEO) satellite’s downlink signals, such as Orbcomm, Iridium-Next, Globalstar, Starlink, OneWeb, SpaceX, etc. The Doppler positioning methods, as one of the main localization algorithms, need a highly accurate receiver design to track the Doppler as a measurement for Extended Kalman Filter (EKF)-based positioning. In this paper, the designed receiver has been used to acquire and track the Doppler shifts of two different kinds of LEO constellations. The extracted Doppler shifts of one Iridium-Next satellite as a burst-based simplex downlink signal and two Orbcomm satellites as continuous signals are considered. Also, with having the Two-Line Element (TLE) for each satellite, the position, and orbital elements of each satellite are known. Finally, the accuracy of the designed receiver is validated using an EKF-based stationary positioning algorithm with an adaptive measurement matrix. Satellite detection and Doppler tracking results are analyzed for each satellite. The positioning results for a stationary receiver showed an accuracy of about 132 m, which means 72% accuracy advancements compared to single constellation positioning.

## 1. Introduction

Navigation using Signals of Opportunity (Nav-SOP) has shown enormous potential as an alternative positioning method in Global Navigation Satellite System (GNSS)-challengeable situations. Integration of Inertial Navigation System (INS) and Global Positioning System (GPS) has been one of the most reliable navigation methods in recent decades. This method utilizes the short-term accuracy of INS and long-term robustness of GPS at the same time. However, some substantial drawbacks of GPS/INS integration methods lead the researchers to focus on the Nav-SOP method, alternatively. Some of the main problems of GPS/INS integration are the possibility of signal interferences, being sensitive to jamming, and inaccessibility in some dense urban and indoor environments. Also, GPS can be blocked or banned in military situations.

**Figure 1:**
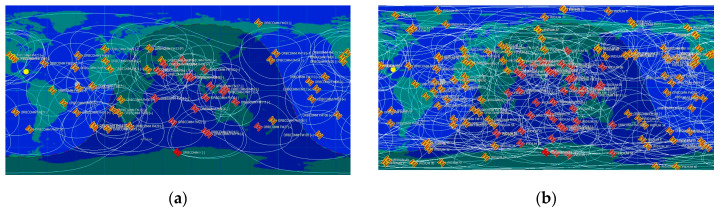
(**a**) Coverage of the Orbcomm constellation in global world map; (**b**) coverage of the Orbcomm and Iridium-Next constellations on a global world map.

There are several kinds of SOPs in different frequency ranges between 3 kHz and 300 GHz in categories of Very High Frequency (VHF), L-band, S-band, Ka-band, etc. These signals freely exist in the environment, also, their accessibility and wide-band frequency range make them the best choices for non-GPS navigation methods. FM, TV, Wi-Fi, DAB-radio, Long-term Evolution (LTE) signals, cellular, and LEO satellite signals are considered as SOPs. Compared to Geosynchronous Equatorial Orbit (GEO) and Medium Earth Orbit (MEO) satellites, LEO satellites are closer to the earth with a more powerful signal and different constellations. Positioning using LEO satellite signals depends on the signal coverage and duration of visibility. Each LEO satellite can be visible to a receiver for a special duration, therefore, after this visible time, the receiver needs to wait until the next satellites is observable. Accordingly, for some constellations, there are times when no satellites are visible to the receiver. Designing a multi-constellation LEO satellite receiver can solve the satellite’s observability problem. Also, it can detect the best Doppler measurement for EKF-based Doppler positioning methods by selecting the suitable measurement model. Figure 1 demonstrates the difference of coverage between a single constellation (Orbcomm) and multi-constellation (Orbcomm/Iridium-Next). It can be seen that by increasing the number of constellations and the observability time, providing continuous Doppler measurements for EKF-based positioning systems can be guaranteed.

This paper presents an adaptive design for a software-defined multi-constellation receiver with the ability to detect the visible satellites and injecting the suitable Doppler measurement to EKF-based positioning systems. Firstly, the method will detect the overhead satellites by performing Welch Power Spectral Density (PSD) analysis, which is called the acquisition part. In the second part, the Doppler frequency of each detected satellite will be tracked with a Phase Locked Loop (PLL) algorithm. Finally, a measurement switching filter is designed for selecting the right measurement for Doppler positioning architecture. The results showed that the receiver could track the Doppler and Doppler rate of 2 satellites at the same time, also, the results for positioning of stationary receiver demonstrated the accuracy improvement compared to the single receiver design.

In Section 2 we present a literature review of previous methods and studies in detail. In Section 3 we describe the EKF-based multi-constellation Doppler positioning method. In Section 4 we define the LEO signal model and receiver design. Experimental results are presented in the Section 5 and finally, the conclusion and potential of future studies are discussed in Section 6.

## 2. Related Works

There are several efforts on addressing the Nav-SOP problems for receiver design and positioning algorithms. In [1,2], the Doppler measurements were obtained from Quadrature Phase-Shift Keying (QPSK) signals of LEO satellites by using carrier synchronization and signal processing design. An opportunistic positioning method using LTE downlink signals was designed, also, a Software-Defined Receiver (SDR) was implemented for extracting and tracking the pseudorange rate measurement from LTE signals in [3]. Likewise, the integration of LTE signals and Inertial Measurement Unit (IMU) was considered in a navigation framework for ground autonomous vehicles [4]. SOP-aided Inertial Navigation Systems (INS) have experimented in various GNSS-challengeable positioning applications. Estimating the location of unknown terrestrial SOPs was performed by adaptive maximum likelihood filter [5], and extended Kalman filter [6]. Observation of SOP’s pseudorange measurements with Time-of-Arrival (TOA) and Time-Difference-of-Arrival (TDOA) was presented for navigation of collaborative flight vehicles [7,8,9].

The SDR designs were studied deeply in [10], however; it completely depended on the GNSS signal observations. In [11], the LEO movement imaging was described by designing a low-earth orbiting radar, which could improve the investigating potential of scientists for studying LEO satellites. Furthermore, the augmentation of GNSS and LEO satellites was analyzed in [12], as a source of space-based monitoring and useful information for Nav-SOP applications. Many positioning methods were studied with some kind of LEO satellite, namely, Doppler positioning with Orbcomm satellites [13], Iridium satellites [14], and Iridium positioning in weak signal environments [15]. These methods could improve the positioning precision in the short-term, however; due to the short visibility time duration of each satellite, the long-term robustness is still under serious investigation.

The instant positioning using the single Globalstar LEO satellite presents the importance of communication between a user terminal and an LEO satellite [16]. Positioning methods based on Doppler measurements are studied with two different estimation algorithms. First, using a nonlinear least-square filter, and second, EKF estimation systems, which are studied in [17,18], respectively. A novel K-band receiver front-end is designed and investigated in [19]. The receiver was presented for inter-satellite communication between LEO and GEO. Their design has had a significant impact on reducing power leakage, hardware cost, and automatic gain calibration. In [20], a method is studied for navigation using carrier phase measurements of LEO satellite constellations. In this paper, the Integer Least-Squared (ILS) problem is defined as a major challenge to be reduced in size and finally, the authors succeeded to decrease it, outstandingly. The fast convergence of an LEO satellite clock was studied in [21], which showed the method for the estimation of the satellite clock in real-time mode.

There were also some efforts to design a Software-Defined Receiver (SDR) for obtaining the Doppler shift measurements of LEO satellites. In [22], an SDR receiver is presented for satellite communication, also, the Satellite’s Doppler, code rate, and code phase search were studied. The authors in [23] have shown a new algorithm for estimating the downlink Doppler frequency of LEO satellites as the main measurements of Doppler positioning systems in Nav-SOP applications. Moreover, the analysis of using Doppler and Doppler rate of LEO satellites for Radio Frequency (RF) geolocation and enhancement of Doppler positioning algorithms for stand-off trajectories are studied in [24,25], respectively. A Precise Point Positioning (PPP) system was also designed and validated in [26,27]. Finally, in [28], a receiver was designed for Orbcomm LEO satellites, which can track the Doppler shift and perform the positioning using a single LEO satellite. Although these designs could improve the accuracy for state estimation of the receiver and LEO satellite, all their experiments are extremely dependent on the time in which the receiver can extract the Doppler measurement from acquired signals. Consequently, designing the multi-constellation receiver will not only expand the measurement’s tracking time but can also enhance the accuracy of Doppler positioning in more functional time.

## 3. Doppler Positioning EKF Model and Parameters

The pseudorange rate measurements were obtained from the LEO receiver in each time-step k for each n visible satellites. This measurement is related to the Doppler frequency with Equation (1). ${\hat{f}}_{d_{n}}\left(k\right)$ is the Doppler frequency of the n−th LEO satellite in time-step k. $f_{c_{n}}$ is the downlink center frequency of n−th satellite, and c is the speed of light. Subsequently, the pseudorange rate measurement of each satellite is defined as in Equation (2),

(1) $$z_{n}\left(k\right)=c\frac{{\hat{f}}_{d_{n}}\left(k\right)}{f_{c_{n}}},$$

(2) $$z_{n}\left(k\right)=\frac{{\dot{p}}_{t,n}{}^{T}\left(k\right)\left[p_{r}-p_{t,n}\left(k\right)\right]}{∥p_{r}-p_{t,n}\left(k\right)∥}+c\left(\dot{\delta }t_{r}-\dot{\delta }t_{t,n}\right)+\varepsilon_{t,n}\left(k\right),$$

where $p_{r}$ is the 3D position vector of the receiver, and $p_{t,n}\left(k\right)$ is the position vector of the n−th satellite. Also, $\dot{\delta }t_{r}$ and $\dot{\delta }t_{t,n}$ are the clock drifts of the receiver and satellite, respectively. It should be mentioned that the ionospheric and tropospheric delays were omitted because of their negligible amounts in each measurement step [29]. As the receiver is assumed stationary, we can suppose that the clock drifts of the LEO satellites and the receiver do not change with time. Therefore, the state and measurement vectors are defined in Equations (3) and (4).

(3) $$x\left(k\right)={\left[\begin{array}{cc} p_{r}^{T} & \begin{array}{cc} \dot{\delta }t_{r} & \begin{array}{cc} \dot{\delta }t_{t,1} & \begin{array}{cc} \ldots & \dot{\delta }t_{t,n} \end{array} \end{array} \end{array} \end{array}\right]}^{T},$$

(4) $$z\left(k\right)={\left[\begin{array}{cc} z_{1}\left(k\right) & \begin{array}{cc} \ldots & z_{n}\left(k\right) \end{array} \end{array}\right]}^{T},$$

As the transition matrix is obtained as an (n+4) dimension identity matrix, the time-update equations of EKF for states vector and error covariance matrix follow $x_{k}\left(k+1\right)=x_{k}\left(k\right)$ and $P_{k}\left(K+1\right)=P_{k}\left(k\right)+Q$, respectively. The EKF equations are presented in Equations (5) and (7) where *Q* is the process noise covariance, *R* is the observation noise covariance, and *K* is the standard Kalman gain.

(5) $$K\left(k+1\right)=P_{k}\left(k+1\right)H^{T}\left(k+1\right){\left[H\left(k+1\right)P_{k}\left(k+1\right)H^{T}\left(k+1\right)+R\left(k+1\right)\right]}^{-1},$$

(6) $$x_{k+1}\left(k+1\right)=x_{k}\left(k+1\right)+K\left(k+1\right)\left[z\left(k+1\right)-H\left(k+1\right)x_{k}\left(k+1\right)\right],$$

(7) $$H\left(k+1\right)=\left[\begin{array}{cc} h\left(k+1\right) & \begin{array}{cc} c.I_{n\times 1} & -c.I_{n\times n} \end{array} \end{array}\right],$$

Herein, H is the measurement matrix and I is the identity matrices, defined by its indices. For a better understanding of the measurement Jacobian matrix, its expanded form is defined in Equations (8) and (9).

(8) $$h\left(k+1\right)={\left[\begin{array}{cc} h_{1}^{T}\left(k+1\right) & \begin{array}{cc} \ldots & h_{n}^{T}\left(k+1\right) \end{array} \end{array}\right]}^{T},$$

(9) $$H\left(k+1\right)={\left[\begin{array}{cccccc} h_{1}\left(k+1\right) & c & -c & 0 & \cdots & 0\\ h_{2}\left(k+1\right) & c & 0 & -c & \cdots & 0\\ \vdots & \vdots & 0 & 0 & \ddots & 0\\ h_{n}\left(k+1\right) & c & 0 & 0 & \cdots & -c \end{array}\right]}_{n\times \left(n+4\right)},$$

Where $h_{n}\left(k+1\right)$ vector is defined as Equations (10) and (11). It should be mentioned that the velocity of the stationary receiver was zero vector.

(10) $$h_{n}^{T}\left(k+1\right)=-h_{v_{t,n}}^{T}\left(k+1\right)+h_{p_{t,n}}^{T}\left(k+1\right).h_{v_{t,n}}\left(k+1\right).h_{p_{t,n}}^{T}\left(k+1\right),$$

(11) $$h_{p_{t,n}}\left(k+1\right)=\frac{p_{r}-p_{t,n}\left(k+1\right)}{∥p_{r}-p_{t,n}\left(k+1\right)∥},\;h_{v_{t,n}}\left(k+1\right)=\frac{-{\dot{p}}_{t,n}\left(k+1\right)}{∥p_{r}-p_{t,n}\left(k+1\right)∥},$$

Finally, after combining Equations (10) and (11), the complete form of $h_{n}^{T}\left(k+1\right)$ is obtained as Equation (12).

(12) $$h_{n}^{T}\left(k+1\right)=\frac{{\dot{p}}_{t,n}\left(k+1\right)}{∥p_{r}-p_{t,n}\left(k+1\right)∥}-\left(p_{r}-p_{t,n}\left(k+1\right)\right).\frac{{\dot{p}}_{t,n}{}^{T}\left(k+1\right)\left[p_{r}-p_{t,n}\left(k+1\right)\right]}{{∥p_{r}-p_{t,n}\left(k+1\right)∥}^{3}}$$

By considering the velocity of the receiver, the mentioned EKF equations can be expanded for a dynamic receiver in mobile ground or flight vehicles. The measurement noise was modeled as zero-mean random white Gaussian with variance $\sigma^{2}$. Also, the initial value of the covariance matrix P(0) was selected with the noise variance on its diagonals. A small positive value was selected for covariance of process and measurement noise matrix.

**Figure 2:**
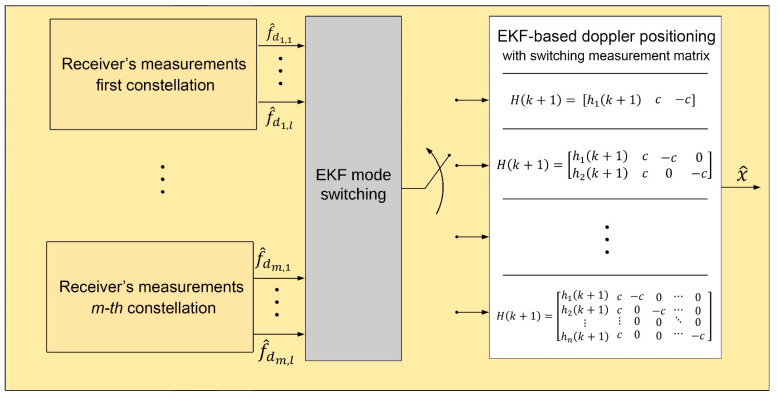
Architecture of proposed multi-constellation LEO satellite receiver.

Figure 2 illustrates the switching mode for EKF-based Doppler positioning. In the proposed system, the type and dimension of the measurement matrix is determined by an EKF mode switching block. After obtaining all the Doppler frequencies from the receiver, the mentioned switching block selects the adapted EKF system by sending the 0 or 1 binary values to the determined EKF system. As each satellite is invisible after a while, the number of measurements may vary with time. As a result of this, the system completely depends on the satellite visibility and the number of measurements. The EKF mode switching block tracks the measurements and modifies the EKF system.

## 4. Multi-Constellation Receiver Design

### 4.1. Characteristics of LEO Downlink Signals

The main concentration of this paper is on two constellations modulated by the QPSK method, namely, Iridium-Next and Orbcomm LEO satellites. These satellites are from different organizations and they were made for communication applications, like voice, fax, data, personal massaging, military, etc. Table 1 consists of several details about each satellite including the number of working satellites in the constellation, number of orbital planes, inclination, height, orbital time period, and downlink frequency range. Also, the Globalstar satellite is added for more comparison. Orbital data of each satellite in all three constellations is updated every day in a TLE file, and it is publicly downloadable from the North American Aerospace Defense Command (NORAD) site, and data is represented with simplified perturbations models (SGP, SGP4, and SGP8).

**Figure 3:**
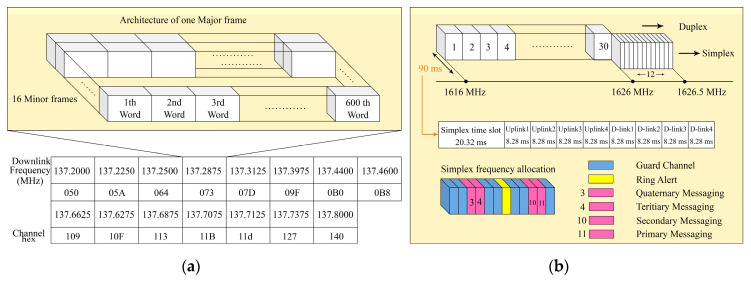
(**a**) Structure of Orbcomm downlink signals and (**b**) architecture of Iridium-Next downlink signals.

The Orbcomm satellites transmit the continuous packed data at a VHF bandwidth frequency between 137 and 138 MHz. The Orbcomm downlink data is transmitted with a Bit rate of 4800 bps. The downlink signal consists of 600 words and each word is 8 bits of data. Each minor frame is 4800 bits and is transmitted in 1 s, also 16 minor frames are defined as one major frame, as depicted in Figure 3. Our last experiments in signal acquisition showed that not all the Orbcomm satellites were functional. Eleven channels transmitted downlink signals and each satellite transmitted in two downlink frequencies. The main difference between Orbcomm and Iridium is that, unlike the Orbcomm which transmits the continuous downlink signals, the Iridium is based on the bursts. This signal’s variety can assess the adaptivity of the designed multi-constellation receiver [30,31].

The Iridium worked at an L-band downlink frequency of 1616 to 1626.5 MHz in both duplex and simplex channels. The Iridium signals were functional in 30 duplex sub-bands between 1616 and 1626 MHz, and 12 simplex channels between 1626 and 1626.5 MHz, which consisted of a 90 ms time division multiples access (TDMA) frame. Both simplex and duplex signals were bursts. It is more common to use the simplex bursts for Nav-SOP applications due to the fact that they are always transmitting from Iridium satellites. As it is demonstrated in Figure 3, simplex bursts were transmitting at different frequencies, which were allocated for Ring alerts and messaging channels. In each simplex timeslot, there were four messaging channels (Primary, Secondary, Tertiary, and Quaternary), one Ring alert, and seven guard channels. The center frequency of the Iridium and Iridium-Next Ring alert was 1626.27833 MHz and center frequencies of messaging channels were 1626.437500, 1626.395833, 1626.145833, and 1626.104167 MHz, respectively [30,31]. The receiver sampled the obtained discretized signal with a special sampling frequency $f_{c}$, and $T=f_{c}^{-1}$ is the sampling time. Due to the fact that the LEO satellite’s downlink signals were transmitted with a QPSK modulator, the receiver signal from all L channels of m−th constellation can be formulated as Equations (13) and (14),

(13) $$R_{m}\left[n\right]=\overset{L}{\underset{l=1}{\sum }}\left|A_{m,l}\right|e^{j\left(k\frac{\pi }{2}+\frac{\pi }{4}\right)}e^{j(\omega_{m.l}n+\varphi_{m.l})}=\overset{L}{\underset{l=1}{\sum }}r_{m,l}\left[n\right],\;k\in \left\{0,1,2,3\right\},$$

(14) $$r_{m,l}\left[n\right]=\left|A_{m,l}\right|\;.\exp\left[j\left(k\frac{\pi }{2}+\frac{\pi }{4}\right)\right]\;.\exp\left[j2\pi nT\left(f_{d_{m,l}}\left[n\right]+f_{{IF}_{m,l}}\right)+j\varphi_{m.l}\left[n\right]\right]+b\left[n\right],$$

where k is the transmitted QPSK signal’s samples, n is the number of samples and t=t(0)+nT is the time of the received signal. $f_{{IF}_{m,l}}$ and $f_{d_{m,l}}$ are intermediate and Doppler frequencies for l−th channel of m−th constellation, respectively. $A_{m,l}$ is the totall power magnitude of the received signal. Also, b[n] is a complex model of zero-mean white Gaussian noise. The $\varphi_{m.l}$ and $\omega_{m.l}$ are acquired phase and angular frequency of the transmitted signal, respectively.

### 4.2. Receiver’s Architecture

**Figure 4:**
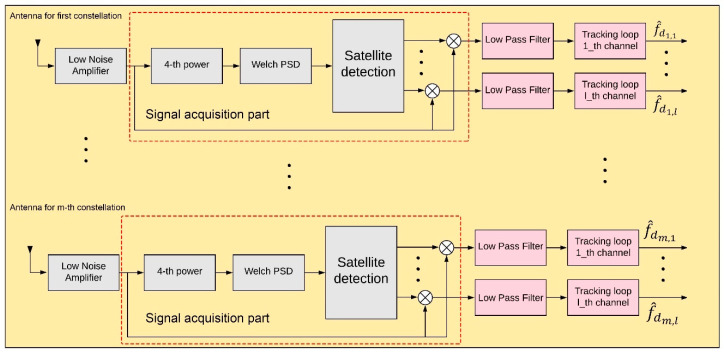
Architecture of proposed multi-constellation LEO satellite receiver.

The main concentration of this paper is on three constellations, which are modulated by the QPSK method, namely, Globalstar, Iridium, and Orbcomm LEO satellites. In this part, the structure of the multi-constellation receiver is presented in two sections. At first, the LEO signal is acquired and detected and in the second part, the Doppler measurement is extracted and tracked to be injected in the EKF-based Doppler positioning system. Figure 4 demonstrates the proposed architecture for both parts of the receiver.

#### 4.2.1. Signal Acquisition

After receiving the signal from the antenna, it goes through the Low Noise Amplifier (LNA) electronic device. This device magnifies the signal with the least change in the signal-to-noise ratio (SNR), also, in the situation that the signal has low-power, it has a significant impact on the accuracy of signal acquisition. The Welch method is a kind of periodogram averaging technique to analyze the Power Spectral Density (PSD) of the received signal [29]. The signal, $R_{m}\left[n\right]$, is divided into K batches and each batch has M number of points. Also, the S is the number of points for overlapping the segments. In Equation (15), a Discrete Fourier Transform (DFT) window, $FR_{m,k}\left[v\right]$, is calculated in each segment at the determined frequencies,

(15) $$FR_{m,k}\left[v\right]=\;\overset{M+\left(k-1\right)S-1}{\underset{n=\left(k-1\right)S}{\sum }}R_{m}\left[n\right]w\left[n\right]e^{-j2\pi vn},\;k=1\;to\;K$$

where, v is the window frequency, and w[n] is defined as the Hamming window function. Finally, the periodogram value was calculated for each segment. Subsequently, the mean value of the periodogram was obtained. Equations (16) and (17) show the estimation of PSD analysis.

(16) $$PP_{m,k}\left[v\right]=\;\frac{1}{G}{\left|FR_{m}\left[v\right]\right|}^{2},\;G=\overset{M}{\underset{n=0}{\sum }}w^{2}\left[n\right]$$

(17) $$PSD_{m,k}\left[v\right]=\;\frac{1}{K}\overset{K}{\underset{k=1}{\sum }}PP_{m,k}\left[v\right]$$

Herein, $PSD_{m,k}\left[v\right]$ is the PSD value for each segment of m−th constellation [20]. Afterward, the satellite detection block finds the peaks of PSD values with a searching window of ±1 kHz around the carrier center frequency. The center frequency of the searching window for each constellation is the middle of its frequency range. The satellite detection block finds the peaks of PSD values and determines the related Doppler frequency. In another view, for burst-based constellations, the satellite detection block will be transferred to the burst detection block in which, the center frequency of each burst in one timeslot is considered. The detected frequencies of peak points were compared to the specific threshold, which was assigned −40 dB. Equation (18) presents the peak finder function for the satellite detection block.

(18) $$\bar{\bar{IF}}=\;\mathrm{Max}\left(PSD_{m,k}\left[v\right]\right)$$

(19) $$U\left(x\right)=\{\begin{array}{cc} \exp\left(-2j\pi f_{\bar{\bar{IF}}}nT\right),\; & \overset{=}{O}\geq -40\;\mathrm{dB}\\ 0, & \overset{=}{O}<-40\;\mathrm{dB} \end{array}$$

The signal U(x) is the output of the satellite detection block, which by multiplying to the main received signal, removes the detected carrier frequency of the signal at the l−th channel. The given signal goes through the Low-Pass Filter (LPF) with a bandwidth of between 0.5 and 1.5 kHz. The LPF’s bandwidth should be determined as a way that the Doppler shift could pass it.

#### 4.2.2. Doppler Tracking System

To extract the Doppler shift in all sampling times, it needs to be tracked by a tracking loop filter. The Doppler shift can be obtained from QPSK signals by using the classical Costas loop. The received signal is multiplied by U(x). The obtained signal followed by an LPF is defined as input of the tracking loop. Equation (20) presents the acquired $S_{m,l}\left[n\right]\;$for l−th channel of the m−th constellation.

(20) $$S_{m,l}\left[n\right]=\left|A_{m,l}\right|\;.\exp\left[j\left(k\frac{\pi }{2}+\frac{\pi }{4}\right)\right]\;.\exp\left[j2\pi nT\left(f_{d_{m,l}}\left[n\right]\right)+j\varphi_{m.l}\left[n\right]\right]+b\left[n\right],$$

**Figure 5:**
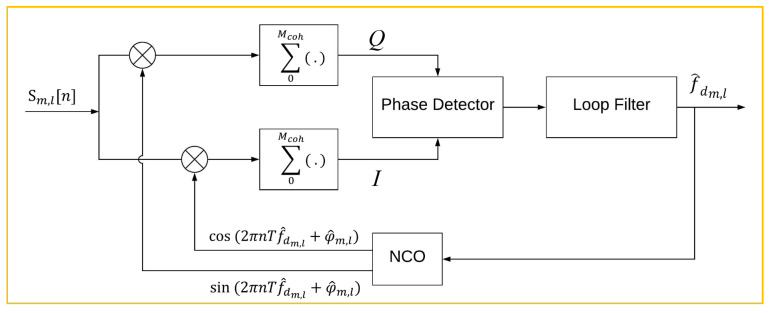
Architecture of proposed multi-constellation LEO satellite receiver.

Figure 5 shows the structure of the tracking loop for each channel of one constellation. It consists of a phase detector, loop filter, Numerically Controlled Oscillator (NCO), and integration functions. The estimated Doppler shift was tracked by a loop filter and NCO accounts for maintaining the detected phase. To obtain the phase residual, the input signal, $S_{m,l}\left[n\right]$, was multiplied by cosine and sine functions of the estimated Doppler and phase. Subsequently, I and *Q* signals were calculated by the sum of the $M_{coh}$ samples of $S_{1}$ and $S_{2}$, which is the integration in continuous mode between time steps *n* and *n* + 1.

Equations (21) and (22) present the obtained *I* and *Q* signals as In-phase and Quadrature components of the signal. $b_{Q}\left[n\right]$ and $b_{I}\left[n\right]$ are the zero-mean white Gaussian noise related to each component. Therefore, the phase discriminator computes the residual phase in each time step with Equation (23).

(21) $$Q=\overset{M_{coh}}{\underset{0}{\sum }}S_{m,l}\left[n\right].\sin\left(2\pi nT{\hat{f}}_{d_{m,l}}\left[n\right]+{\hat{\varphi }}_{m.l}\left[n\right]\right)\approx \left|A_{Q}\right|\sin\left(\delta {\hat{\varphi }}_{m.l}\left[n\right]\right)+\;b_{Q}\left[n\right],$$

(22) $$I=\overset{M_{coh}}{\underset{0}{\sum }}S_{m,l}\left[n\right].\cos\left(2\pi nT{\hat{f}}_{d_{m,l}}\left[n\right]+{\hat{\varphi }}_{m.l}\left[n\right]\right)\approx \left|A_{I}\right|\cos\left(\delta {\hat{\varphi }}_{m.l}\left[n\right]\right)+b_{I}\left[n\right],$$

(23) $$\delta {\hat{\varphi }}_{m.l}\left[n\right]=\frac{\left|A_{I}\right|}{\left|A_{Q}\right|}.\mathrm{atan}\left(\frac{Q}{I}\right),$$

The first-order loop filter tracks the Doppler shift with continuous transfer function F(s). Also, the phase is tracked by the NCO with the transfer function of G(s). Equations (24) and (25) show the transfer functions and H(s) is the transfer function of the closed-loop system.

(24) $$F\left(s\right)=\frac{as+1}{bs},\;G\left(s\right)=\frac{K_{NCO}}{s},$$

(25) $$H\left(s\right)=\frac{F\left(s\right)G\left(s\right)}{1+F\left(s\right)G\left(s\right)}=\;\frac{2\xi \omega_{n}s+\omega_{n}{}^{2}}{s^{2}+2\xi \omega_{n}s+\omega_{n}{}^{2}\;},\;\omega_{n}=\;\frac{2B_{l}\xi }{\xi^{2}+0.25\;},$$

Where, $\omega_{n}$ and ξ are obtained from NCO’s gain ($K_{NCO}$) and parameters of loop filter (a, b), known as loop natural frequency and damping ratio, respectively. The value of ξ = 0.707 is selected to have a flat response, also, the $\omega_{n}$ can be selected with the noise bandwidth $B_{l}$ [32]. The NCO block updates the phase using the estimated phase and Doppler shift of the previous time step. The phase is updated due to Equation (26).

(26) $${\hat{\varphi }}_{m.l}\left[n\right]=\;{\hat{\varphi }}_{m.l}\left[n-1\right]+\;2\pi \left(n-1\right)M_{coh}{\hat{f}}_{d_{m,l}}\left[n-1\right],\;{\hat{\varphi }}_{m.l}\left[0\right]=0,$$

Signal-to-Noise Ratio (SNR) for l−th channel of m−th constellation is defined as $SNR_{m,l}=\frac{A_{m,l}.T_{coh}}{N_{0}}$, where $T_{coh}$ is the sampling time of Q and I signals for the phase detector block. Also,$\;N_{0}$ is the PSD value of the transmitted signal’s noise. The noise variance of tracking loop block for l−th channel of the m−th constellation can be estimated as $\sigma_{PLL,m,l}^{2}=2\sigma_{\varphi ,m,l}^{2}.B_{l}.T_{coh}$, where noise variance of the phase detector, $\sigma_{\varphi ,m,l}^{2}$, is defined in Equation (27) [33]. Finally, the noise variance of the receiver’s Doppler measurements are obtained as $\sigma_{LEO,m,l}^{2}=\;{\left(\frac{c}{f_{c}}.\sigma_{PLL,m,l}\right)}^{2}$, where *c* is the speed of light.

(27) $$\sigma_{\varphi ,m,l}^{2}=\;SNR_{m,l}^{-1}\left(\frac{8}{9}SNR_{m,l}^{-6}+\frac{20}{3}SNR_{m,l}^{-5}+\frac{10}{3}SNR_{m,l}^{-4}-\frac{8}{3}SNR_{m,l}^{-3}+2\;SNR_{m,l}^{-2}\right)$$

## 5. Experimental Results

**Figure 6:**
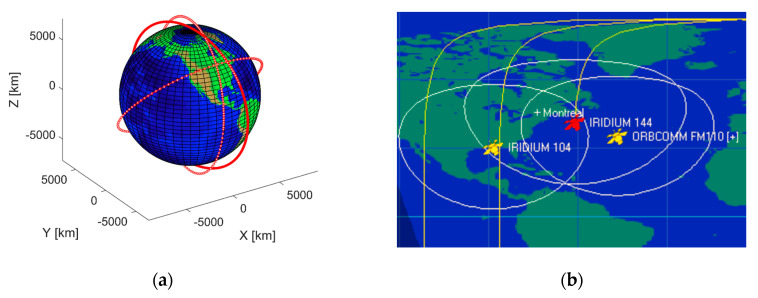
(**a**) Position of satellites during one orbital period and (**b**) the location of satellites over Montreal’s region.

**Figure 7:**
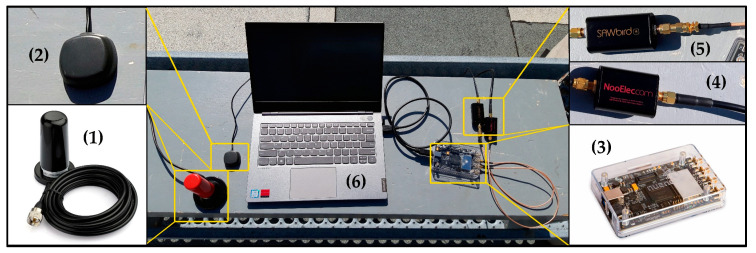
(**1**) Dual VHF/UHF antenna for Orbcomm constellation; (**2**) Iridium antenna; (**3**) BladeRF V2 SDR; (**4**) RF bandpass filter; (**5**) Low-Noise Amplifier (LNA) for Iridium frequency range; (**6**) PC.

The designed multi-constellation receiver was tested by recording the samples of two visible constellations, Iridium-Next and Orbcomm with a sampling frequency of 2 MHz. The experiment was performed on the roof of the ETS university in downtown Montreal City with BladeRF Multi-Input Multi-Output (MIMO) Software Defined Radio (SDR), which used for recording the data from visible satellites. One channel of SDR was allocated for Orbcomm with center frequency of 137.5 MHz, and the other channel was determined for Iridium simplex bursts with center frequency of 1626.25 MHz. A dual-band VHF/UHF mobile antenna was utilized for Orbcomm signals, in parallel, an Iridium antenna was selected for the second channel. During the experiment, one Orbcomm satellite (ORBCOMM FM110), and two Iridium-Next satellites (IRIDIUM 104, IRIDIUM 144) were detected overhead. TLE files of all three satellites were downloaded, and their positions were tracked and simulated by an open-source SGP4 simulator, as depicted in Figure 6. It was completely necessary to use an RF bandpass filter for the Orbcomm channel, and an ultra-Low-Noise Amplifier (LNA) for the Iridium channel. Figure 7 shows all the utilized equipment in this experiment.

**Figure 8:**
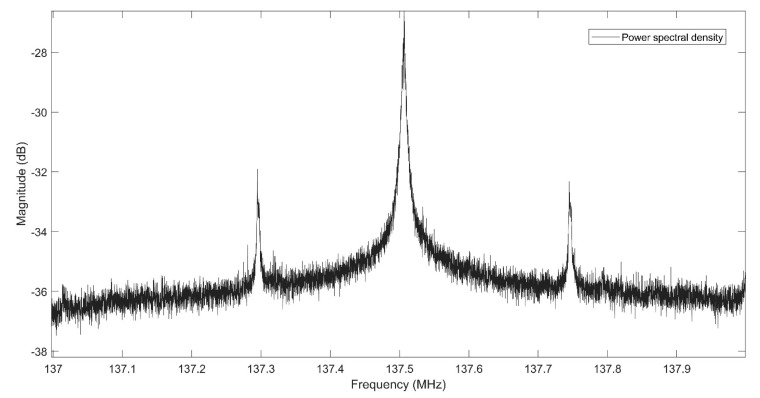
Power Spectral Density (PSD) analysis of acquired Orbcomm signal.

**Figure 9:**
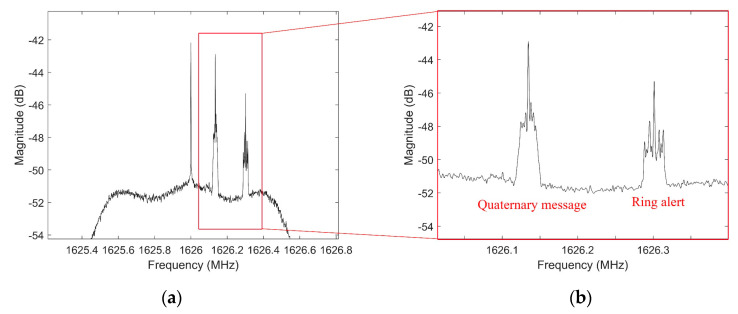
(**a**) PSD of Quaternary message burst of Iridium-Next and (**b**) PSD of Ring alert burst of Iridium-Next.

The BladeRF has its own embedded RF front-end, so the provided LNA can be sufficient for the signal acquisition of Iridium-Next bursts. Using one multi-channel SDR made the system more adaptive for changing the center frequencies and sampling times of receiving signals. Although the BladeRF would not be able to record two far sampling frequencies at the same time, it can change the center frequency rapidly with negligible delay. At the time of experiment, one Orbcomm satellite (Orbcomm FM-110) with downlink transmitting center frequencies of 137.2875 and 137.7375 MHz was visible. Also, two Iridium-Next satellites were detected overhead. The Welch Fast Fourier Transform (FFT) power spectral density analysis was implemented for recorded Orbcomm samples, as depicted in Figure 8. Also, Figure 9 shows the PSD analysis of one Ring alert and one Quaternary message bursts of Iridium-Next before burst frequency filtering. The peak frequency of the Quaternary message was detected as 1626.1346 MHz, and the frequency of 1626.3013 MHz was the FFT peak of the Ring alert burst.

**Figure 10:**
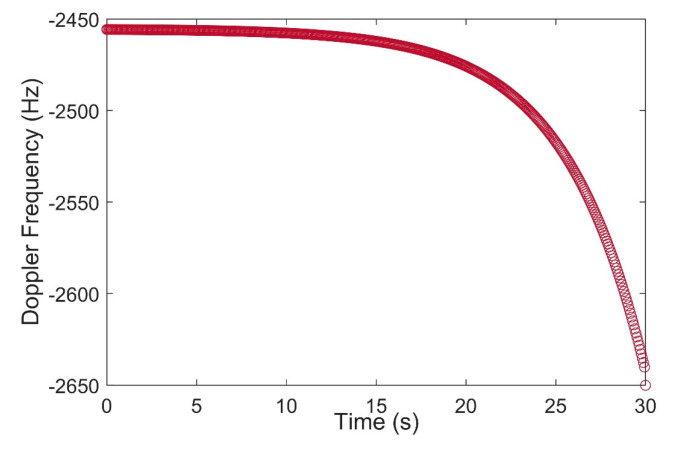
Doppler shift tracking for Orbcomm FM110 in 30 s measurements.

The average bandwidth of bursts for Ring alert and Quaternary messages were between 30 and 35 kHz. Furthermore, message bursts had a higher magnitude compared to the Ring alerts. The Doppler tracking loop for each satellite can be initialized by a simple calculation of the difference between the obtained and determined frequencies. Also, the Doppler tracking loop for the Iridium constellation was continuous for each burst’s duration. As a result of this, the initial Doppler value for Ring alert and message bursts were 30.32 and 30.30 kHz for Iridium 104, and 29.55 and 29.62 kHz for Iridium 144, respectively. Likewise, for Orbcomm constellation, the lower band frequency was considered for positioning with an initial Doppler value of approximately −2.4564 kHz. Figure 10 shows the result of the Doppler tracking block after Orbcomm signal acquisition over a duration of 30 s. The results were used as the pseudorange rate measurements of the Orbcomm constellation for Doppler positioning.

**Figure 11:**
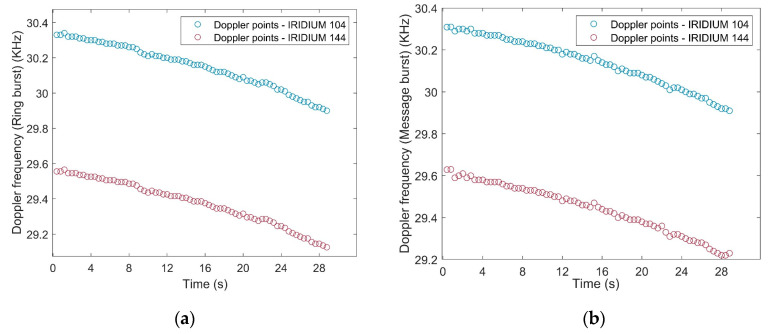
Doppler points for two Iridium satellites using (**a**) Ring alert bursts and (**b**) Quaternary message bursts.

Also, Figure 11 demonstrates the extracted Doppler shift points from Iridium’s simplex bursts. The duration of each simplex signal was 90 ms [30], so approximately 72 Doppler points were attained after 30 s of the experiment for both Ring alert and Quaternary message bursts. The Doppler tracking system tracked the Doppler shift in each burst duration and finally, each point was calculated as the average of Doppler in 90 ms. The measurement of the Orbcomm constellation was continuous and in contrast, the measurement of the Iridium constellation was based on the burst durations. The Interpolation method was used for the Doppler measurements of Iridium constellations, followed by the same sampling frequency for synchronizing the measurements of both constellations.

**Figure 12:**
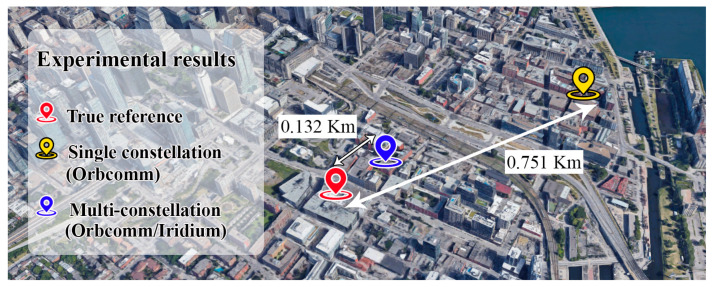
Positioning results for a stationary receiver using single-constellation (Orbcomm) and Multi-constellation (Orbcomm and Iridium-Next) receiver modes.

The proposed EKF-based Doppler positioning method was implemented and tested in MATLAB, using the obtained Doppler shift measurements of the designed multi-constellation software-defined receiver. The Doppler measurements of one Orbcomm satellite and two Iridium-Next satellites were injected into the positioning system. The experiment was performed one time using Ring alert, and the other time using Quaternary message bursts. Moreover, the positioning result is compared to a single constellation mode. Figure 12 demonstrates the estimated and true reference position for a stationary receiver.

The estimated positions were obtained using continuous Doppler measurements of Orbcomm and points measurements of Ring alert and messaging bursts for Iridium-Next satellites. The initial value for the north-direction and east-direction position axes of the receiver were chosen about 20 km away from the true reference. Also, by knowing the height of the building (roof of the ETS university), where we carried out the experiment, the initial value of the altitude axis was selected with a 100 m difference. Table 2 shows the results of the designed Doppler positioning using single and multi-constellation methods. According to Table 2, the positioning using measurements of a multi-constellation receiver was more precise in messaging burst mode than Ring alert burst mode.

The messaging bursts usually have a more powerful magnitude, which can be a more useful signal for positioning. However, the results have shown significant improvement compared to the single constellation mode using both Ring alert and messaging bursts. As no altimeter was used in this experiment, the altitude estimation is the most challengeable issue, however; using an accurate altimeter or initializing the positioning system with true altitude, can expressively increase the system’s precision. As a result, the 3D Root Mean Square (RMS) for messaging burst mode is about 177 m compared to the real location, which shows about 76% accuracy advancement compared to the single constellation method. Also, this RMS value using Ring alert bursts is about 213 m with 72% improvement with no altimeter aiding.

## 6. Discussion

The principal perspective of this research was to design and present a novel adaptive architecture for most types of LEO satellite receivers in multi-constellation mode. The Doppler frequency, clock bias, and clock drift of satellites are known as the main measurements in Doppler positioning algorithms. The designed measurement model can be selected alternatively, with regards to the number of satellites and constellations, also, burst and continuous downlinks are considered in the proposed architecture. The importance of LEO satellite receivers can be proved when the accuracy of measurements for Doppler positioning methods is being investigated. In the continuous mode of downlink signals, like the Orbcomm constellation, the receiver utilizes the Doppler tracking mode by detecting the center frequency of overhead satellites. In contrast, for the burst-based constellations, like Iridium, the bursts should be detected and tracked, which leads to a discrete Doppler trajectory.

**Figure 13:**
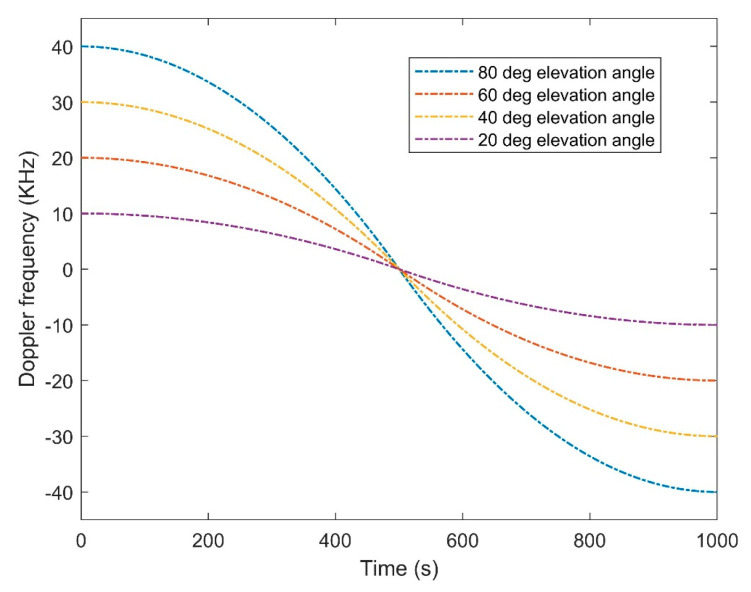
Approximation of the Doppler curve for LEO downlink signals in different elevation angles.

The operated SDR in the proposed experiment is BladeRF 2.0 micro with a frequency range of 47 MHz to 6 GHz and the ability of automatic gain control and automatic IQ and DC offset correction. It should be discussed that because of a significant difference between the center frequency of two used constellations, the BladeRF should be used in switch mode for changing the center frequency between two constellations. Figure 13 presents the Doppler curve of each downlink signal of LEO satellites during the same pass time of the satellite. The higher Doppler frequency means a higher elevation angle between the satellite and the receiver. As the satellite approaches the receiver’s location, the Doppler will tend to zero, oppositely, negative Doppler shows the inverse direction and movement away from the receiver.

It should be mentioned that the complete curve will be obtained when the satellite passes through the receiver. The experiment in this research estimated the Doppler shift from recorded data over a duration of 30 s. This time duration was selected for two main reasons. First, visibility time for Orbcomm satellites is usually between 1 and 2 min, in contrast, Iridium-Next satellites in outdoor environments are visible most of the time. Furthermore, to perform the experiment in multi-constellation mode, the duration should be selected in a way that both constellations be visible. Second, using huge amounts of measurements over a long duration cannot assess the accuracy of the receiver in static mode. To obtain the static location of the receiver, using less measurements can validate the accuracy of the proposed method and its capability for real-time implementations.

The method does not have any augmentation like a GPS reference initializer or altimeter. As the main purpose of this research is the design and validation of presented multi-constellation LEO-SDR receiver, the validation was performed in static mode to show the improvement of obtained measurements in a simple Doppler localization system. As a result, in the Ring alert burst mode, the RMS error of positioning decreased from 765 to 213 m, likewise, in messaging bursts mode, the error reached 132 m in a no-augmentation situation. The receiver also showed moderate improvement in altitude error.

## 7. Conclusions

In this paper, an adaptive multi-constellation software-defined LEO satellite receiver is designed and implemented. The receiver’s architecture is compatible to be used for Doppler positioning with LEO satellite SOPs. Nav-SOP has been under investigation by researchers as an important alternative of GNSS navigation, in recent decades. However, coverage and visibility of LEO satellites over a long-term duration are the key issues of using this kind of navigation. Designing a multi-constellation receiver is the first step to utilize the pseudorange rate measurements of various LEO satellites. It can make the navigation more trustable for flight and ground vehicles, due to its dependency on the measurements of various constellations. Also, Nav-SOP can be integrated with INS to cope with GNSS-challengeable environments or situations, in which the GNSS is inaccessible.

In the proposed architecture, the multiple antennas can be used for acquiring the LEO signals from different constellations. For more than two satellites, it is essential to use more SDRs for recording the data at the same time with different center frequencies. The receiver is designed to detect the satellites transmitting center frequency and also to recognize the burst for burst-based satellites like Iridium. The Doppler tracking is managed to follow the Doppler measurements with special sampling frequency for all kinds of LEO satellites. The accuracy of Doppler positioning using measurements of the proposed receiver is completely dependent on initial values and EKF parameters.

The system can be useful in LEO-INS integration applications, collaborative multi-constellation navigation designs, and timing systems. It is an open research issue to investigate the design of a tracking loop for burst-based satellites to obtain more accurate measurements. Moreover, the EKF can be designed to estimate the position and velocity of LEO satellites, as well as the receiver’s states. The dependency of the system on the downloaded orbital information from the TLE file leads to a basic error due to the inaccuracy of LEO information from TLE files. Apart from that, the positioning for dynamic flight and ground vehicles can be studied in multi-constellation modes.

Although the designed receiver showed acceptable results for stationary receivers, using an altimeter or other aiding measurements is expected to improve the positioning accuracy, especially in-flight dynamic experiments. In addition, a deep comparison between the results of the architecture using various loop filters and phase discriminators can provide future research topics. The designed system can be used in experiments of GNSS-SOP-INS integration with a GNSS cut-off time in dynamic and static situations for drones, Unmanned Aerial Vehicles (UAV), ground vehicles, and submarine unmanned vehicles. Finally, in some cases, the feasibility of LEO-SOPs for enhancing the heading precision can be studied in many indoor and outdoor positioning and localization systems.

## Figures and Tables

**Table 1:** Specifications of Orbcomm, Globalstar, and Iridium LEO satellites.

	Working Satellites	Orbital Planes	Inclination	Height	Orbital Period	Frequency Range
Globalstar	48	8	52°	1414 km	~113 min	2483.5~2500 MHz
Iridium	66	6	86.4°	781 km	~100 min	1616~1626.5 MHz
Orbcomm	30	6	45°, 70°	825 km	~99 min	137~138 MHz

**Table 2:** Comparison between Root Mean Square (RMS) error of each method.

	2D RMS	3D RMS	Altitude RMS	Mode
Single Constellation (Orbcomm)	0.751 km	0.7658 km	0.150 km	Continuous
Multi-Constellation (Orbcomm + Iridium)	0.176 km	0.2136 km	0.121 km	Ring alert bursts
	0.132 km	0.1771 km	0.118 km	Messaging bursts
